# The differing responses of central carbon cycle metabolism in male and female *Sargassum thunbergii* to ultraviolet-B radiation

**DOI:** 10.3389/fpls.2022.904943

**Published:** 2022-10-03

**Authors:** Yan Sun, Yu Zang, Jun Chen, Shuai Shang, Jing Wang, Qian Liu, Xuexi Tang

**Affiliations:** ^1^ College of Marine Life Sciences, Ocean University of China, Qingdao, China; ^2^ Laboratory for Marine Ecology and Environmental Science, Qingdao National Laboratory for Marine Science and Technology, Qingdao, China; ^3^ Key Laboratory of Marine Eco-Environmental Science and Technology, First Institute of Oceanography, Ministry of Natural Resources, Qingdao, China; ^4^ College of Biological and Environmental Engineering, Binzhou University, Binzhou, China

**Keywords:** *Sargassum thunbergii*, central carbon cycle metabolism, metabolites, diecious differences, UV-B radiation

## Abstract

The enhancement of ultraviolet-B radiation (UV-B) radiation reaching the Earth’s surface due to ozone layer depletion is an important topic. Macroalgal species growing in the intertidal zone are often directly exposed to UV-B radiation periodically as the tide changes. In order to better understand the response of macroalgae to UV-B stressed condition, we studied the dominant dioecious intertidal macroalgae *Sargassum thunbergii*. After consecutive UV-B radiation treatments, we used metabonomics models to analyze and compare the maximum photosynthetic electron transport rate (ETR_max_), central carbon cycle metabolism (CCCM) gene expression level, CCCM enzymic activities [pyruvate dehydrogenase and citrate synthase (PDH and CS)], and carbon-based metabolite (including pyruvate, soluble sugar, total amino acid, and lipids) content in male and female *S. thunbergii*. The results showed that under low and high UV-B radiation, the ETR_max_ values and six targeted CCCM gene expression levels were significantly higher in males than in females. Under high UV-B radiation, only the CS activity was significantly higher in males than in females. There was no significant difference in PDH activity between males and females. The CCCM models constructed using the metabonomics analysis demonstrate that *S. thunbergii* males and females exhibit obvious gender differences in their responses to UV-B radiation, providing us with a new understanding of the macroalgal gender differences under UV-B radiation, as past investigations always underestimated their diecious characteristics.

## Introduction

Solar radiation strongly affects photoautotrophic organisms worldwide. It drives photosynthesis, which transforms CO_2_ to carbohydrate and releases O_2_ (Mckenzie et al., 2007; Fraikin, 2018). A considerable amount of global primary production results from the photosynthesis of intertidal macroalgae under photosynthetically active radiation (PAR, 400–700 nm). However, these macroalgae also experience stress from ultraviolet radiation (UVR, 280–400 nm) (Gao et al., 2007). Over the past several decades, the radiation levels reaching the Earth’s surface have increased due to depletion of the stratospheric ozone. This increase in radiation affects human economic, scientific, and technological activities (Staehelin et al., 2001). UVR ranges from 200 to 400 nm. It is sub-divided into UV-A (315–400 nm), UV-B (280–315 nm), and UV-C (200–280 nm) radiation. It has been demonstrated that UV-A radiation generally does not influence the normal metabolic activity of organisms. UV-C is known to be fully absorbed by ozone (Zlatev et al., 2012). Although UV-B accounts for only 1.5% of the total spectrum of solar radiation reaching the surface of the Earth, that energy consists of high-energy photons that affect photosynthetic organisms in both terrestrial and aquatic habitats (Hanelt, 1998; Caldwell et al., 2007; Wijewardana et al., 2016). The diverse negative effects and defense mechanisms associated with UV-B have been well documented in terrestrial ecosystems (Ueda and Nakamura, 2011; Carlos, 2014). The responses of intertidal macroalgae to high levels of UV-B could provide new perspectives.

Approximately 20,000 known species of macroalgae or seaweed are distributed in intertidal zones around the world (Critcheley and Ohno, 1998). In addition to fixing carbon (known as “Blue Carbon”) (Nellemann et al., 2009), macroalgae also serve as a spawning, nursery, and feeding ground for marine organisms and control nutrient cycling in coastal ecosystems. Because of their commercial values, only a small number of macroalgal species have been fully characterized and investigated. However, from the perspective of ecological potential, macroalgae play an important role in their corresponding ecological niches. Since most macroalgae are distributed in intertidal zones and upper subtidal zones, they are affected by different levels of UVR as the tides change (Häder et al., 2011). As a key factor influencing the vertical distribution of intertidal macroalgae (Zou and Gao, 2009; Jiang et al., 2018), UVR in intertidal areas is much higher than that in terrestrial areas, meaning that carbon metabolism in intertidal macroalgal species is directly exposed to UV-B. It is noteworthy that their physiological responses also have been influenced by UV-B stresses.

**Figure 1:**
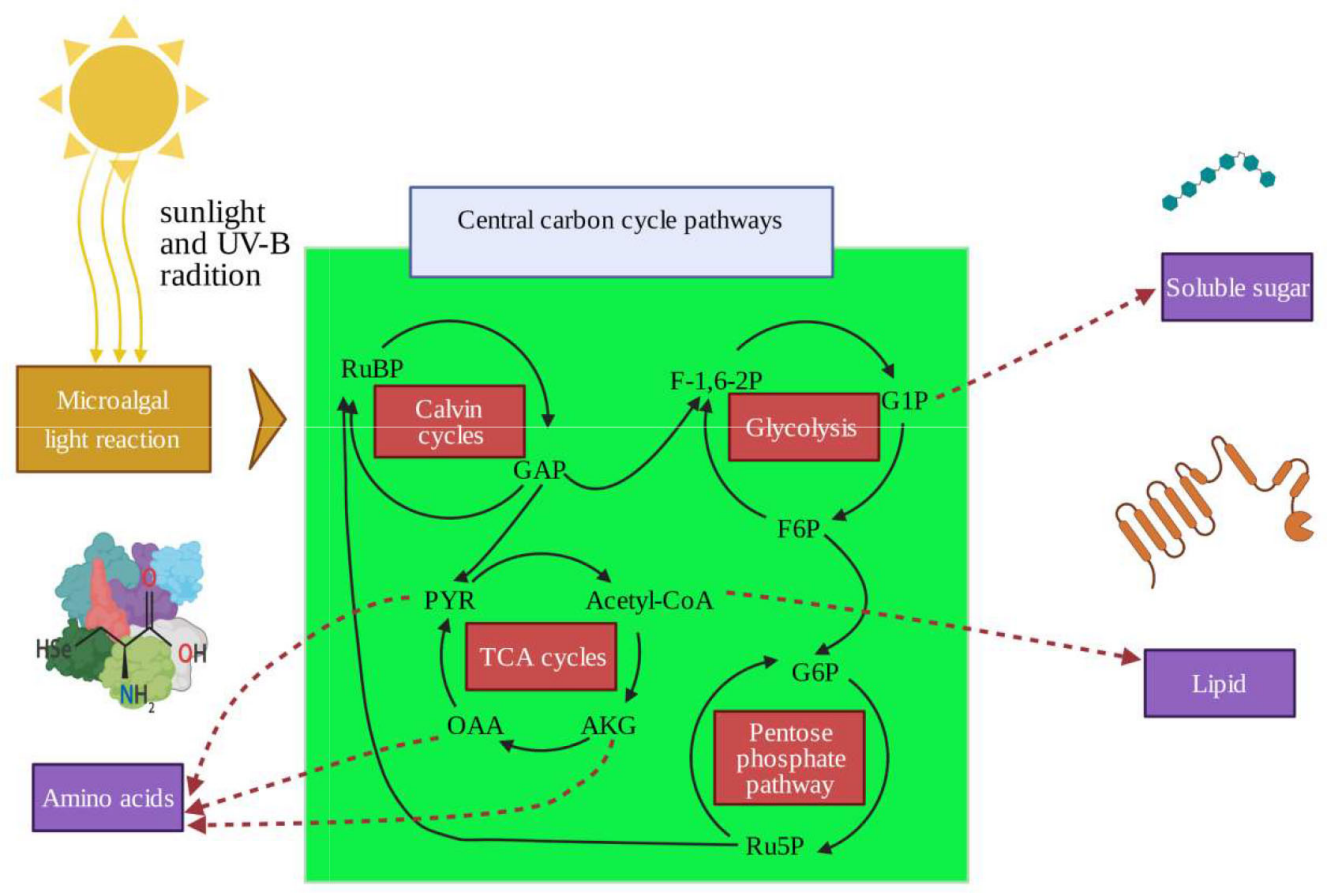
Central carbon cycle metabolism and the production of soluble sugar, amino acids, and lipids. RuBP, ribulose-1,5-bisphosphate, GAP, glyceraldehyde-3-phosphate, PYR, pyruvate; AKG, α-ketoglutaric acid; OAA, oxaloacetic acid; F-1;6-2P, fructose 1, 6-bisphosphate; G1P, glucose-1-phosphate; F6P, fructose-6-phosphate; G6P, glucose-6-phosphate; Ru5P, d-xylulose 5-phosphate.

Changes in morphology, physiological metabolic activity, and growth in response to enhanced UV-B have been well characterized in land plants (Jansen et al., 1998; Fedina and Velitchkova, 2009; Zedek et al., 2021). Growth rates, photosynthetic efficiencies, nutrition uptake rates, and reproductive process are inhibited. Secondary metabolite (mycosporine-like amino acids, flavonoids, polysaccharides) biosynthesis, anti-oxidation reactions, and damage to genetic material also occur in high UV-B radiation treated experiments (Jenkins, 2009; Fierro et al., 2014; Wu et al., 2021). In order to moderate UV-B stress, aquatic plants or seaweeds have developed their own specific adaptive mechanisms (Hader et al., 2014). For example, physiological responses in macroalgae under UV-B stresses, including the accumulation of UV radiation-absorbing compounds, antioxidant components, and repair mechanisms, have been investigated in previous research (Yakovleva and Titlyanov, 2001; Heo and Jeon, 2009; Lee and Shiu, 2009). In basic metabolic process, the carbon fixation metabolism (Calvin cycle), tricarboxylic acid metabolism (TCA), glycolysis (embden meyerhof pathway, EMP), and pentose phosphate metabolism pathways constitute the central carbon cycle metabolism (CCCM) (Johnson and Alric, 2013; Obata et al., 2013). Driven by the photoreaction process and reducing force of NADPH, CCCM could determine and allocate the flow of carbon and alter metabolite production (**Figure 1**). The changes in macroalgae metabolites are an important indicator response to UV-B radiation and are also closely related to the stress resistance capacity (Fu et al., 2021; Xue et al., 2022).

Seaweeds are classified into three groups based on pigmentation, i.e., brown (*Phaeophyceae*), red (*Rhodophyceae*), and green algae (*Chlorophyceae*). *Sargassum thunbergii* (Sargassaceae, Phaeophyta) is a brown intertidal dioecious seaweed that forms seaweed beds along the northwestern Pacific coast. Seaweed beds have high economic and ecological value (Park et al., 2005; Liu et al., 2012). Previous studies have indicated that *S. thunbergii* have specific photoprotective mechanisms, reactive oxygen scavenging enzymatic systems, and are highly tolerant to the thermal, osmotic, and desiccation stresses of intertidal environments (Roleda et al., 2007; Kim et al., 2010). Several studies have compared differences among adult, early, and germling life stages of *S. thunbergii* (Chu et al., 2011). However, few reports have compared the characteristic adaptation responses to environmental stresses in dioecious seaweeds. Additionally, research on terrestrial dioecious plants has revealed that it is more burdensome to maintain population stability under environmental stresses for in dioecious plants than for plants without sexual dimorphism. As a result of the dual challenges of human activities and global environmental changes, the resources of macroalgae have been seriously attenuated. Some species are even tending towards being endangered. Therefore, it is imperative to identify dioecious macroalgae and carry out the differentiation treatment in our researches. Our study investigates the CCCM response to high intensity UV-B radiation in male and female *S. thunbergii*. Using previous experimental results and metabonomics analysis, our objective was to analyze and compare differences between male and female *S. thunbergii* exposed to UV-B, focusing on the expression of several key carbon metabolic genes, enzymic activity, and the level of carbonaceous metabolite biosynthesis.

## Materials and methods

### Macroalgae collection and culturing

Fertile female and male *S. thunbergii* were collected from the intertidal zone of the Spring Bay of Qingdao (36°02’58.3’’N, 120°21’31.9’’E) in 2021. The laboratory culture of *S. thunbergii* followed the procedures in our previous studies (Sun et al., 2022). The macroalgae were gently rinsed with sterilized seawater to prevent microbial contamination. The macroalgae received 150 μmol photons·m^−2^·s^−1^ PAR irradiance over 12:12 h of light/dark period at 20 ± 0.5°C. The culture seawater was sterilized and continuously renewed every day until macroalgal samples were collected.

### UV-B radiation treatments and metabolomic analysis

After 10 days of habituated culturing, healthy *S. thunbergii* thalli were separated into female and male specimens and randomly assigned to three groups according to different irradiation intensities. For the UV-B radiation dose, we referred to previous studies and used the UV-B real time detection system in Qingdao coastal areas (Software Copyright: 2018SR747573). The results of our daily monitoring showed that during the reproductive stage of *S. thunbergii* (June–August), the average UV-B radiation intensity in Qingdao from 10:00 to 14:00 was 2.29 W·m^−2^·s^−1^, and the maximum instantaneous intensity could reach 3.7 W·m^−2^·s^−1^. From 6:00 to 18:00 the average UV-B radiation intensity was no more than 1.2 W·m^−2^·s^−1^. According to these data, the high and low UV-B radiation levels in our experiments were set to 2.5 and 1 W·m^−2^·s^−1^, respectively. PAR was set as the normal illumination (control group); PAR with 1 W·m^−2^·s^−1^ UV-B radiation was the low intensity group; and PAR with 2.5 W·m^−2^·s^−1^ UV-B radiation was the high intensity group. The radiation treatment duration was 12 h per day. There were six replicates of each experimental group. After 3 days of treatment, 80 mg of thalli samples was quickly weighed, ground, and stored in liquid nitrogen at −80°C. The samples underwent metabonomics analysis using liquid chromatography-mass spectrometry (LC/MS) equipped with an electrospray ionization (ESI) source. The samples were separated with an Agilent 1290 Infinity LC ultra-high performance liquid chromatography system (UHPLC) and a hydrophilic interaction liquid chromatography column (HILIC). They were then analyzed with a Triple TOF 5600 mass spectrometer (AB SCIEX, DH Tech. Dev. Pte. Ltd., MA, USA). The data were subjected to one-way ANOVA to evaluate differences in the metabolites among treatments, with a significance level of *p* < 0.05.

### Profiling of rapid light curves

A pulse amplitude-modulated (PAM) fluorometer (Imaging-PAM fluorometer, Walz, Effeltrich, Germany) was used to measure the rapid light curves (RLCs) of male and female *S. thunbergii* macroalgae under different amounts of UV-B radiation. At the beginning of the experiment, the *S. thunbergii* genders were determined and labeled before PAM analysis was carried out. All treatments included PAR/+UV-B radiation exposure (8 h duration) at day 0, 1, 3, and 5.

### Quantitative real-time polymerase chain reaction

In the first step of CCCM, ribulose-l,5-bisphosphate carboxylase/oxygenase (Rubisco) is catalyzed by CO_2_ into glyceraldehyde 3-phosphate (G3P). This process connects carbon fixation with the Calvin cycle and directly affects photosynthetic rates. In the next step, fructose-1,6-bisphosphatase (FBP), citrate synthase (CS), acetyl-CoA carboxylase (ACC), succinic dehydrogenase (SDH), and sorbitol dehydrogenase (SODH) co-worked in CCCM, which were connecting to carbon assimilation and metabolism processes, and as rate-limiting enzymes affect the synthesis and accumulation of subsequent metabolites. Although pyruvate phosphodikinase (PPDK) can dissipate excess light energy in photoautotrophic organisms, it cannot complete the inorganic carbon assimilation process, including the synthesis and extension of carbon skeletons (Dayan et al., 2013). The expressed quantities of seven key central carbon cycle enzyme coding genes were analyzed using RT-qPCR. After 3 days of treatment with PAR (+UV-B), 100 mg of macroalgae was collected and ground. Following the manufacturer’s instructions, total RNA was isolated from the macroalgal cells using TRIzol^®^ (Sigma, Shanghai, China) and One-Step. A gDNA Removal and PrimeScript™ RT reagent Kit with gDNA Eraser (Takara, Dalian, China) was used for reverse transcription (Takara, Dalian, China). After total RNA extraction, agarose gel electrophoresis (1% agarose w/v) and a Picodrop spectrophotometer (Picodrop, Cambridge, UK) were used to determine RNA quality and concentrations. Each sample was analyzed three times. The *18S rRNA* and *β-actin* were selected as reference genes. The genes’ relative expression patterns were analyzed using the 2^-ΔΔCt^ method. The results were presented as target gene fold changes in the treated group divided by fold changes in the control group.

### Metabolite content and enzymatic activity determination

After 3 days of treatment with PAR (+UV-B), 500 mg of macroalgae was collected and ground for metabolite content and enzymatic activity determination. Appropriate kits A081-1-1 (Jiancheng Bioengineering Institute, Nanjing, China) and spectrophotometric methods were used to determine the amount pyruvic acid, which was based on its reaction with 2,4-dinitrophenylhydrazine to form pyruvic acid-2,4-dinitrophenylhydrazine appearing red in alkaline solution. The amount of pyruvic acid in the sample can be calculated by measuring the absorbance of the sample at 520 nm. Soluble sugar, total amino acids, and lipids are the key carbon skeleton metabolites participating in macroalgal physiological activity and stress resistance (Tziveleka et al., 2021). The amount of these metabolites in the thalli of *S. thunbergii* have been determined in previous research (Sun et al., 2022) and their proportion were calculated.

Appropriate kits (BC0385 and BC1065) (Solarbio Science and Technology Co. Ltd., Beijing, China) were used to test the activity of pyruvate dehydrogenase (PDH) and citrate synthase (CS), respectively. PDH activity detection is based on the dehydrogenation of pyruvate and 2,6-dichlorophenol indigo, detected with 605 nm light absorption. The activity was detected based on the difference before and after the reaction. CS catalyzes acetyl-CoA and oxaloacetic acid to produce citrate co-enzyme A, which is further hydrolyzed to produce citric acid. This reaction causes DTNB to transform into yellow TNB with absorbance at 412 nm.

### Date analysis

Fisher’s exact test was used to perform KEGG (Kyoto Encyclopedia of Genes and Genomes) pathway enrichment analysis and calculate the significance level of metabolite enrichment. The metabolomic data analysis details and parameter settings can be found in previously published research (Sun et al., 2022). The physiological, biochemical, and molecular experimental data are presented as means ± SD (n = 3). The SPSS 20.0 program (SPSS, Chicago, USA) was used for statistical analysis. Different treatments were compared using one-way ANOVA tests, followed by Student’s *t*-tests. Statistical significance was considered at the *p* ≤ 0.05 level. Pearson’s correlation analysis was used to analyze the relationships among ETR_max_, gene expression levels (*Rubisco*, *ppdk*, *sodh*), enzymic activities (PDH and CS) and the metabolites content (soluble sugar, total amino acids, lipids and pyruvate) based on the results of the 3-day experiment in dioecious *S. thunbergii*. Statistical significance was evaluated at the *p* ≤ 0.05 level.

## Results

### Metabolomic results and the analysis of central carbon cycle metabolism

**Figure 2:**
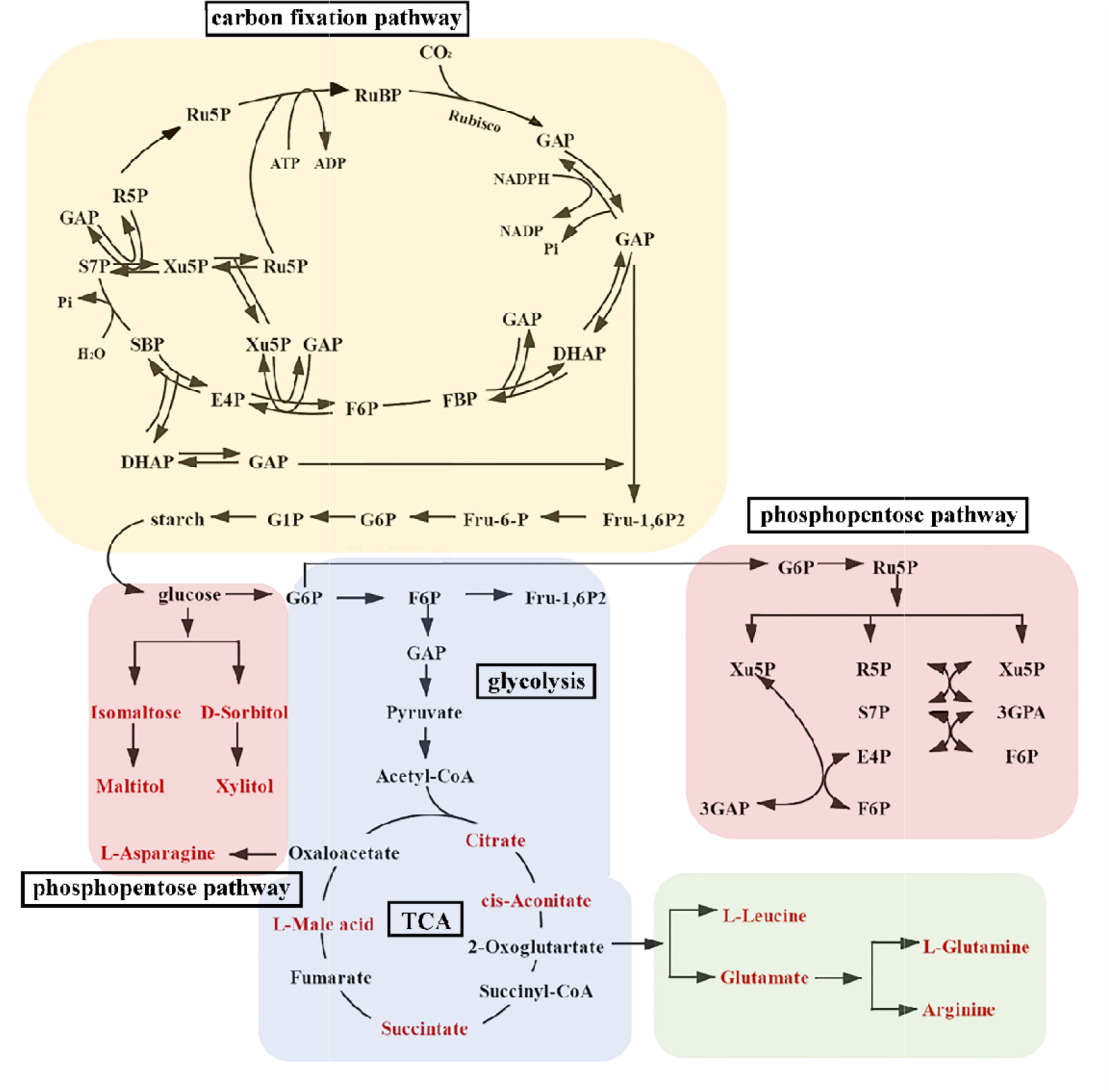
Model of central carbon cycle metabolism in *S. thunbergii.* The red names represent metabolites with significantly differences between female and male algae. The abbreviations for metabolites are: Ru5P, d-xylulose 5-phosphate; RuBP, ribulose-1,5-bisphosphate; 3GAP, glycerate-3-phosphate; GAP, glyceraldehyde-3-phosphate; DHAP, dihydroxyacetone phosphate; FBP, fructose-1,6-diphosphate; F6P, fructose-6-phosphate; E4P, erythrose-4-phosphate; SBP, sedoheptulose-1,7-bisphosphatase; S7P, Sedoheptulose 7-phosphate; Xu5P, xylulose 5-phosphate; R5P, ribose5-phosphate; F-1, 6P2, fructose 1, 6-bisphosphate; Fru-6-P, fructose-6- phosphate; G6P, glucose-6-phosphate; G1P, glucose-1-phosphate.

Overall, there were significant changes in 111 kinds of metabolites. Eighty-four metabolites were significantly regulated under different treatments in male *S. thunbergii*. Thirty-three metabolites were significantly regulated under different treatments in female *S. thunbergii*. Precisely 64 species of significantly regulated metabolites (accounting 57.66% of total regulated species) are present in amino acids, sugar, and nucleotides. These compounds were derived from the carbon fixation process and distributed by the CCCM. In the CCCM model (**Figure 2**), when we compared the amount of metabolites and the male/female ratio values, the significantly different metabolites between males and females (13 species) all presented in the TCA, glycolysis, and pentose phosphate metabolism, and the specific values (LC/MS analyzed data and male/female ratio values) are presented in **Table 1**.

**Table 1:** Significantly different accumulation metabolites in CCCM analyzed by metabolomic results.

Fold change ratio (male: female)	control group	low UV-B radiation treatment	high UV-B radiation treatment
isomaltose	0.48 (30804:64550)	0.35 (38622:109502)	0.08 (10687:136512)
D-sorbitol	0.31(46634:15222)	0.39 (29432:74679)	2.43 (260616:107138)
maltitol	0.59 (137209:231543)	0.27 (76774:284928)	0.28 (59796:213140)
xylitol	0.50 (209765:422227)	0.37 (182556:494492)	0.68 (59578:87688)
L-asparagine	2.28 (292895:128582)	2.87 (275960:96014)	2.59 (335505:129750)
citrate	0.47 (139284:298931)	0.32 (130757:412225)	NA
cis-aconitate	0.53 (97381:185452)	4.15 (447770:107898)	0.55 (48467:88192)
succinate	NA	4.46 (272754:61097)	3.50 (30398:86857)
L-malic acid	0.37 (43184:117598)	NA	NA
L-leucine	1.68 (596682:356035)	4.53 (150202:61097)	5.36 (126184:23632)
glutamate	NA	NA	0.66 (711039:1080492)
L-glutamine	2.47 (9456397:3400562)	1.64 (7510143:4568952)	2.71 (114041:40739)
arginine	0.41 (27671:68025)	NA	0.38 (25516:139342)

(The values in parentheses are from the results of metabolomic in our experiments, as measured by mass spectrometry).

The numbers represent the fold change ratio between male and female S. thunbergii under different conditions. NA indicates there were no significant differences in metabolites between male and female *S. thunbergii*.

The CCCM metabolomic analysis results show that in the control group, low UV-B radiation group, and high UV-B radiation group, eight, five, and six kinds of metabolites, respectively, were significantly higher in female *S. thunbergii* than in males. Therefore, it is not difficult to conclude that UV-B radiation changed the accumulation of metabolites, and that there were gender differences in the respective regulated processes. In the analysis of the dioecious *S. thunbergii* metabolism results, we found that there were obvious gender differences or significant regulatory changes in the amount of cis-aconitate, succinate, L-malic acid, arginine and D-sorbitol, citrate, succinate, L-malic acid, and glutamate under different treatments. In the high UV-B treatment group, the amount of isomaltose was 11.50 times higher in female macroalgae than in males. This was the greatest difference observed in this study. L-leucine is a significant regulatory metabolite in male macroalgae. In the control group, it was 5.87 times higher in males than in females. With intensifying UV-B, L-leucine was 4.53 times higher in males than in females in the low UV-B treatment group and 5.36 times higher in males than in females in the high UV-B treatment group. The content of D-sorbitol, citrate, cis-aconitate, glutamate, and arginine showed obvious regulation, which presented that the original gender difference appearing reversed proportion or no longer had gender difference under the stimulation of UV-B radiation. Their corresponding ratio patterns have apparent reversals, which might indicate that these are key metabolites in the CCCM response to UV-B.

### Rapid light curve results

**Figure 3:**
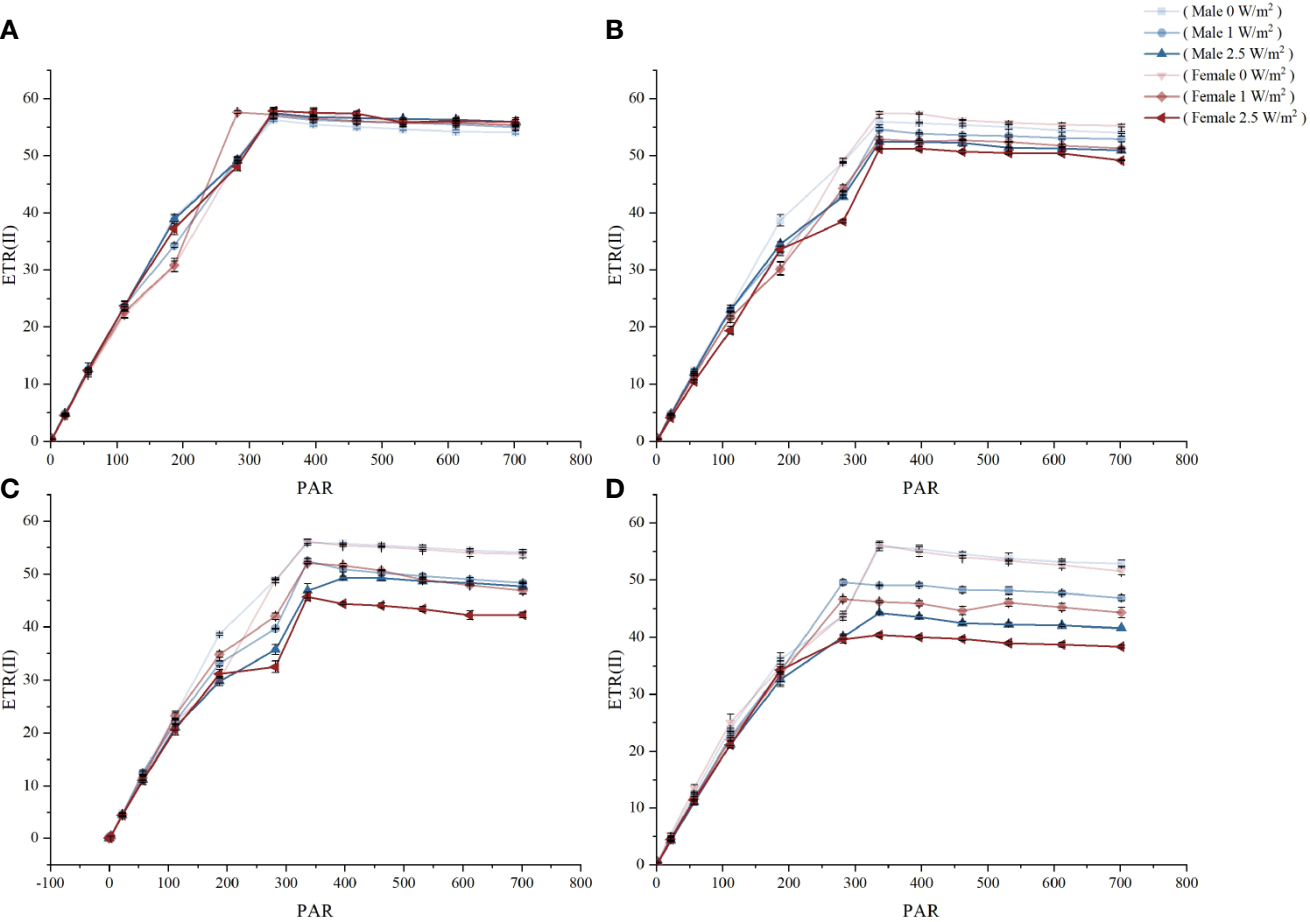
The effects on RLC of different treatments in male and female *S. thunbergii*. **(A)** day 0; **(B)** day 1; **(C)** day 3; **(D)** day 5.

**Figure 3** shows the RLC response of the maximum photosynthetic electron transport rate (ETR_max_) in male and female *S. thunbergii* under different treatments. Before UV-B radiation treatments, there were no significant differences in ETR_max_ between males and females (**Figure 3A**). After 1 day, UV-B radiation had a slight inhibitory effect on ETR_max_, but there was no significant difference between males and females. After 3 days of treatment, only high UV-B radiation caused a significant difference in ETR_max_ (22.7% higher in males than females, *p*<0.05). After 5 days, males had significantly higher amounts than females under low and high UV-B radiation. Although extended time or high intensity UV-B radiation decreased the ETR_max_ values, male *S. thunbergii* always had more stable potential photosynthetic capacity than females.

### Gene expression in the *S. thunbergii* CCCM

**Figure 3** shows the expression patterns of seven CCCM enzyme coding genes (*rubisco*, *fbp*, *ppdk*, *cs*, *sudh*, *acetyl-CoA*, and *sodh)* based on RT-qPCR analysis. The results are shown as the expressed quantities of target genes in the treatment group compared to the expressed quantities in the corresponding control group.

Under low-intensity UV-B radiation, the expression of seven targeted genes was not inhibited in male macroalgae (their expression was higher or the same as in the control group). The *fbp* had the lowest expression levels in females, significantly lower (84.98%) than that in the control group. The greatest change in male macroalgae occurred with the *cs* gene (6.57 times that in the control group). In female macroalgae, the greatest change occurred with the *sodh* gene (17.68 times that in the control group). Low intensity UV-B radiation did not dramatically promote the expression of *sodh* genes in male algae (the difference was slightly higher (36.36%, *p*>0.05) than control group).

Under high-intensity UV-B radiation, the carbon fixation related genes *rubisco*, *ppdk*, and *fbp* were obviously suppressed in female macroalgae. However, they maintained normal transcript levels in males as compared to the control group. With the high-intensity UV-B radiation treatment, the *sodh* gene in female macroalgae had the highest value (21.65 times the controls).

### CCCM-related enzyme activity determination

**Figure 4:**
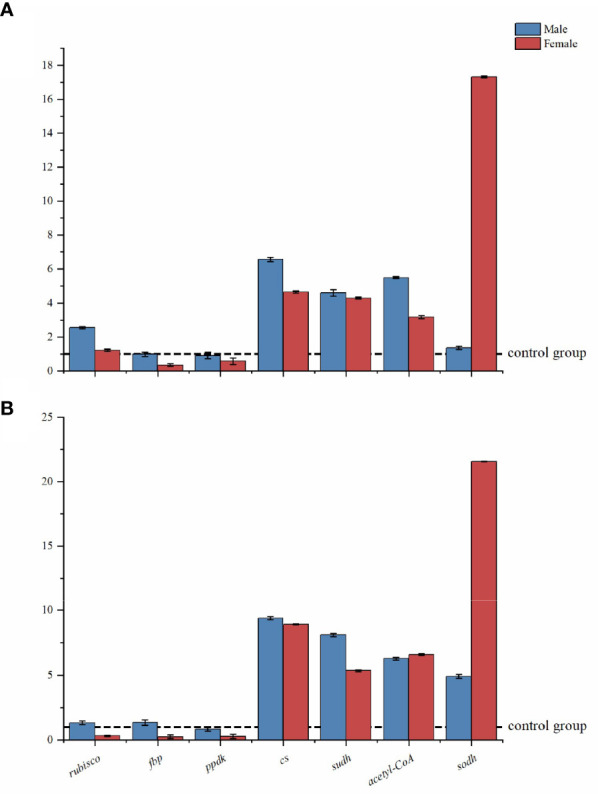
Relative expression levels of CCCM genes in male and female *S. thunbergii* macroalgae under low-intensity **(A)** and high-intensity **(B)** UV-B radiation. The y-axis represents treatment group/control group fold changes. The control group gene expression was set at 1.

The enzymatic activity of PDH and CS in male and female *S. thunbergii* is shown in **Figure 4**. As one pathway in CCCM, PDH and CS connect to the carbon fixation pathway and TCA, which catalyze pyruvate, acetyl-CoA, and oxaloacetate to citrate. With increasing UV-B intensity, this pathway was slightly inhibited in PDH activity in both male and female macroalgae. In the control group, low UV-B radiation group, and high UV-B radiation groups, the activity of PDH was 20.54% (*p*>0.05), 21.76% (*p*>0.05), and 21.72% (*p*>0.05) higher in males than in females respectively. With respect to individual females or males, there was no significant difference in PDH activity in the two UV-B radiation treatments.

CS activity in the control group and the low-intensity UV-B radiation treatment group showed no significant differences between male and female macroalgae. Low-intensity UV-B radiation could stimulate the activity of CS, which was increased by 170.74% and 86.97%, respectively, in males and females compared to the control group. Under high-intensity UV-B radiation, the CS activity was significantly (1.03 times) higher in males than in females. The CS activity in females was similar to that of the control group. CS activity ranged from 14.81 to 27.68 U·g^−1^ (males) and from 12.22 to 34.55 U·g^−1^ (females) in the three treatment groups.

### The proportion and the distribution of carbon-based metabolites

**Figure 5:**
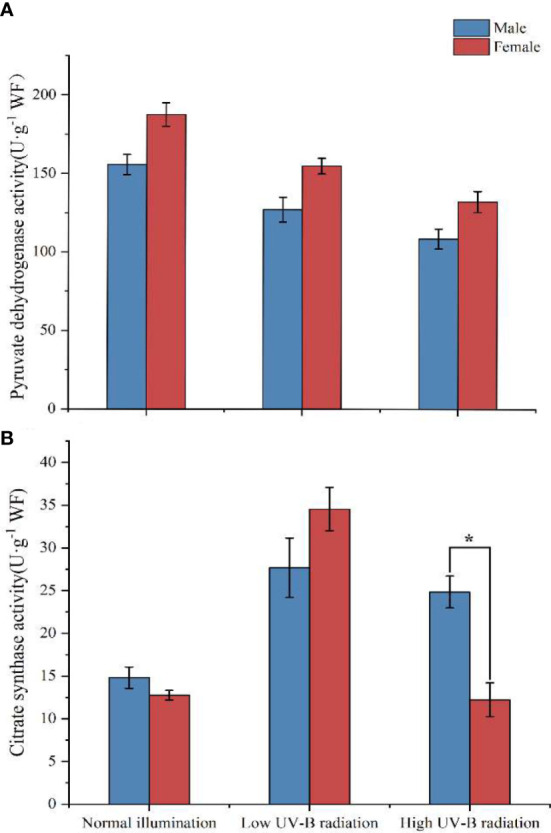
The activities of pyruvate dehydrogenase **(A)** and citrate synthase **(B)** in male and female *S. thunbergii* algaes under different conditions.

Soluble sugar, total amino acids, lipids, and pyruvate in *S. thunbergii* after different treatments were examined after 3 days of UV-B irradiation. There were no significant differences between the amount of pyruvate in male and female *S. thunbergii* in the three experimental groups (**Figure 5**). UV-B radiation did not affect the amount of pyruvate in either gender of macroalgae. This result is consistent with our metabolomic results (**Figure 2**) and demonstrates the high accuracy and stability of metabolomic analysis.

**Figure 6:**
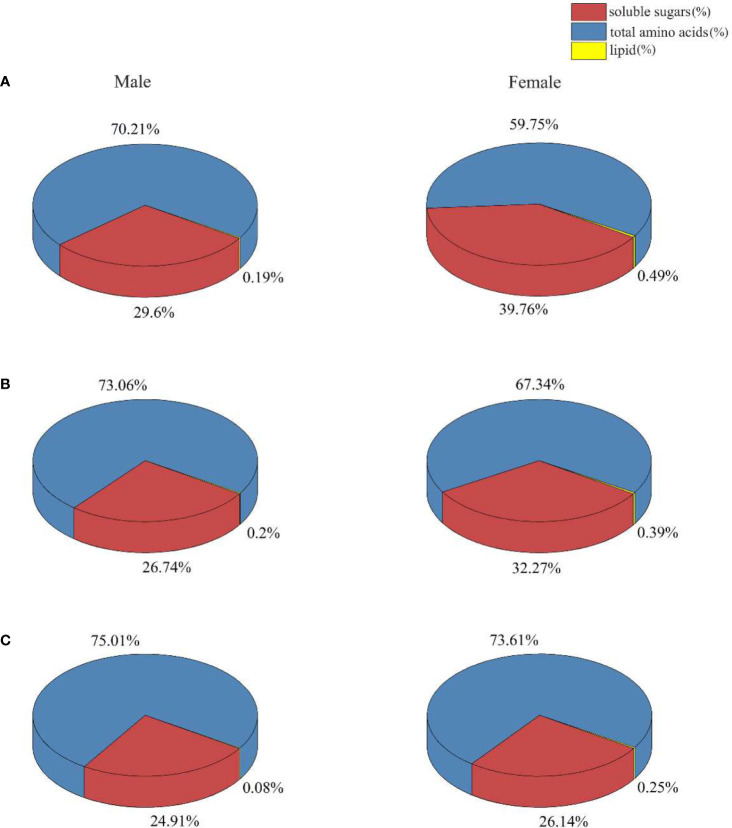
The proportion of soluble sugars, lipids, and amino acids in male and female *S. thunbergii* under different conditions **(A)** control group; **(B)** low UV-B radiation; **(C)** high UV-B radiation.

Proportion and regulation in soluble sugar, total amino acid, and lipid in male and female *S. thunbergii* subjected to different treatments are shown in **Figure 6** and **Table 2**. In the control group, the amount of soluble sugars and total amino acids was higher in males (19.43%, *p*>0.05) than in females (88.54%, *p*<0.05). In contrast, there was a significant difference between male and female macroalgae in the amount of lipids. The amount was higher in females (1.63 times, *p*<0.05) than in males. In the low-intensity UV-B treatment group, the biosynthesis of soluble sugar and total amino acids in females were enhanced as compared to the control group. There were no significant differences in soluble sugar and total amino acid contents. The macroalgal soluble sugar and lipid contents in the females were higher (16.10%, *p*>0.05 and 86.79%, *p*<0.05) than those in the males. The amount of total amino acids was 11.41% lower in females than in males (*p*>0.05). The high-intensity UV-B treatment significantly altered the distribution of all targeted materials between male and female *S. thunbergii*. The lipid content was more than two times higher in females than in males. The amount of sugar and amino acids was significantly higher in males than in females.

**Table 2:** Amount of soluble sugar, total amino acid, and lipid in male and female *S. thunbergii* under different conditions.

Treatment	Gender	Soluble sugar (µmol/mg)	Total amino acid (µmol/mg)	Lipid (µmol/mg)
Control group	Male	7.56 ± 0.64	17.93 ± 2.63*	0.048 ± 0.001*
	Female	6.33 ± 0.46	9.51 ± 0.24*	0.078 ± 0.003*
Low UV-B treatment	Male	7.02 ± 0.52	19.19 ± 0.17	0.053 ± 0.002*
	Female	8.15 ± 0.09	17.00 ± 0.61	0.099 ± 0.012*
High UV-B treatment	Male	7.20 ± 0.35 *	21.69 ± 1.61*	0.022 ± 0.003*
	Female	5.16 ± 0.25 *	14.53 ± 1.08*	0.050 ± 0.005*

Significant differences (p < 0.05) between male and female *S. thunbergii* are indicated by an asterisk (*).

## Discussion

Metabolomic models, photophysiological RLCs, gene expression patterns, enzymatic activity, and the levels of carbon-based metabolites were analyzed to understand changes in CCCM pathway regulated mechanisms and their carbon-based metabolites under UV-B stimulation as well as differences in the responses of male and female *S. thunbergii*.

### CCCM perturbations in dioecious *S. thunbergii* exposed to UV-B radiation based on metabolomic analysis

Different kinds of metabolites were identified and categorized into seven chemical classes, including alcohols, amines, amino acids, fatty acids, inorganic acids, organic acids, and sugars. More than 40 metabolites constituted the CCCM models in **Figure 2**. Even in the control group, a series of metabolites showed significantly different accumulation between males and females. These metabolites perform reproductive functions and support resilience (Obeso, 2002). For example, typical nitrogen substances (L-asparagine, L-leucine, L-glutamine) have high concentrations in male *S. thunbergii* under no stress. Leucine can be used as a source of dissolved organic carbon. Its uptake has been verified to be lower than that of other amino acids (Venolia et al., 2020). In general, the significantly higher amount of leucine in males might mean males have a more direct energy supply for resisting adverse environmental conditions. Leucine can serve as complementary carbon pool in CCCM and provided carbon sources if the carbon fixation processes have been interrupted (Zhang and Bryant, 2011; Liu et al., 2017). The amount of single-stranded carbon materials such as isomaltose, D-sorbitol, maltitol, and xylitol was higher in females than in males. These substances can serve as carbon chain substrates and assemble into complex metabolites, which is reflected in the higher lipid contents in females (**Figure 6**).

Under low-intensity UV-B radiation stress, the amount of cis-aconitate, succinate, L-malic acid, and arginine was higher in males than in females. Under high-intensity UV-B radiation stress, the amount of D-sorbitol, citrate, and succinate was higher in males than in females. Under low-intensity UV-B radiation, all CCCM metabolites remained unchanged compared to the control group, or increased in males. Only glutamate changed significantly, decreasing in males under high-intensity UV-B radiation. From this, we can conclude that male and female *S. thunbergii* have different regulatory mechanisms underlying their response to UV-B stresses. Males depend on a series of metabolites, including carboxylic acid, sugar, and amino acids, in responding to stress. Otherwise, female *S. thunbergii* resisting UV-B stresses might depend on the metabolites of glutamate. Nitrogen-based substances, such as amine and amino acid, also showed differences in male and female *S. thunbergii* and their contents also have regulations under UV-B radiation. Resistance in macroalgae not only reflected dioecious differences, but also differences between carbon (C) and nitrogen (N) metabolism related pathways. Several N-based metabolites constitute the “nitrogen pool” in macroalgae, balancing nitrate reduction, ammonium assimilation, photosynthesis, and protein synthesis (Huovinen et al., 2006; Venolia et al., 2020).

In the CCCM models, providing carbon skeletons for the next carbon metabolism, the carbon fixation processes have unique performances The amount of metabolites in male and female *S. thunbergii* did not change under UV-B stimulation. The gene expression results show that the transcription of the three genes *rubisco*, *ppdk*, and *fbp* was either maintained at normal levels or inhibited in males and females. Under low-intensity UV-B radiation treatment, the expression level of *rubisco* was promoted only in males. Furthermore, gene expression regulation was not influenced by the amount of carbon fixation metabolites. Metabolite biosynthesis might also be involved in the process of functional enzyme and transcript translation. The carbon fixation process might be less vulnerable to UV-B radiation. In carbon fixation pathways, as the source of glycolysis, glyceraldehyde-3-phosphate (G3P) could be guaranteed, especially in male and female *S. thunbergii* stressed by high UV-B radiation. In previous studies, macroalgal photosynthetic capacities and their carbon fixation processes showed direct correlations with marine carbon assimilation so they received more attention than other topics (Cornwall et al., 2011; Krause et al., 2018). In intertidal conditions, some species could regain full photosynthetic potential and perform carbon fixation during tidal cycles. As a typical intertidal species, the upper intertidal macroalgae also exposed to UV-B radiation. Under UV-B radiation, there were no significant differences between male and female *S. thunbergii* in the carbon fixation process related metabolites. This demonstrates that *S. thunbergii* might have stable carbon fixation abilities. Their carbon fixation related metabolites showed no response to UV-B radiation. This not only supports the hypothesis that macroalgae have specific survival strategies in inorganic carbon acquisition but also shows that the macroalgae play a significant role in marine organic carbon storage.

### Photosynthetic characteristics and carbon-based metabolite regulation in dioecious *S. thunbergii* exposed to UV-B radiation

Photosynthesis is the most basic physiological activity in photosynthetic organisms. This process is sensitive to changes in habitat (Häder et al., 2002). In our experiments, ETR_max_ values reflected the transmission of the electron transport chain from the PSII systems to the carbon fixation pathways (Kramer et al., 2004). This provides the reductive and driving force in photosynthesis. ETR_max_ can be inhibited by adverse conditions, including nutrient starvation, adverse temperatures, adverse salinities, and adverse light radiation (Nygård and Dring, 2008; Rautenberger et al., 2013). On prolonged exposure to high-intensity UV-B radiation, ETR_max_ was inhibited to different degrees depending on gender. Based on the photosynthetic ETR_max_ values, males were more UV-B tolerant than females. After three and five days of UV-B radiation treatments at high and low intensities, males and females presented different responses. These differences are consistent with the results of our previous studies, which have confirmed that males have a significantly superiority performance in the chlorophyll fluorescence parameters either low or high UV-B radiation treatments (Sun et al., 2022). The higher photosynthesis activity in males indicates that male *S. thunbergii* has higher UV-B stressed resistance. This higher UV-B stressed resistance not only ensures the stability of the physiological state, but also ensures the supply of CCCM and subsequent carbon skeletons. Compared with males, females performed weakness in the high UV-B radiation resistance and they may used more energy to resist stress and synthesize lipids as well as reflected in the content of soluble sugar and free amino acid lower than the males.

Intertidal macroalgae can use inorganic carbon as a carbon source for carbon-based metabolite energy storage. Under UV-B stresses, dioecious *S. thunbergii* performed regulation at transcriptional and enzymatic levels, which led to different amounts of metabolites. Because of the inherent connection between carbon and nitrogen metabolisms in macroalgae, environmental stress can influence soluble sugar, amino acids, and lipid contents. For example, researchers used stressors such as nutrient starvation and low temperatures to increase the fatty acid composition of different species of algae (Rashid et al., 2014). In our results, the amount of total lipid in female *S. thunbergii* was always higher than in males, in the control group as well as in the UV-B stressed groups. Female *S. thunbergii* were more likely to accumulate lipids than males. This trend is seen in other seaweed species and might be related to the multiplicative process for egg cell formation (Korpelainen, 1992). Previous studies have examined the lipid content and constituents in different macroalgae, such as brown algae *Ecklonia cava* (Altamirano et al., 2003), *Turbinaria ornate*, and *S. mangarevense* (Stiger et al., 2004) and red algae *Gracilaria verrucosa* (Khotimchenko, 2006). These studies often sampled a mixture of juvenile thalli of different genders and attributed their findings to different development stages. In general, higher lipid content is associated with the synthesis of structural components such as membranes. High concentrations of glycolipids and phospholipids in shoots and juveniles may be evidence for rapid growth and development of algae under favorable environmental conditions.

As non-structural carbohydrates, soluble sugars are the main energy source in organisms. They provide carbon skeletons to other metabolites and also play an important regulatory role in plant growth, development, and stress response (Wang and Tang, 2014). In contrast to lipids, soluble sugars can serve as a buffer and a skeleton substance. Soluble sugars can cross membrane structures and transform energy at any time, which can increase the tolerance of plants to abiotic stress and contribute to the osmotic regulation of cells (Sudesh, 2010). Soluble sugars and amino acids also serve as functional molecules for signaling (Koch, 1996). Specifically, sugar signal molecules regulate transcription processes in target genes or organelles. They cause corresponding physiological and biochemical changes, thus regulating the growth and development of plants (Boriboonkaset et al., 2013). Under high-intensity UV-B radiation, soluble sugar concentrations were significantly higher in males than in females. This suggests that males reserved more energy supply and adversity-regulating metabolites to resist damages than females. Thus, the males “carbon skeleton pool” especially for some typically carbohydrate including D-sorbitol, citrate, and succinate brought more strong regulatory abilities.

Under normal conditions and high-intensity UV-B radiation, the amount of total amino acids was significantly higher in male *S. thunbergii* than in females. Under low-intensity UV-B radiation, the amount of total amino acid in females was close to that of males. This finding is similar to that found in land plants in that, generally, males have higher nitrogen concentrations for pollen biosynthesis and a higher nitrogen demand than females (Wang and Griffin, 2003; Zhang et al., 2014). However, it is clear from these studies that females have elevated amino acid levels under low-intensity UV-B radiation, which means that the biosynthesis originating from the glycolytic and gluconeogenesis pathways has been up-regulated. As the soluble sugars, amino acids are major organic nitrogenous compounds involved in the storage and transport of nitrogen. They are also precursors of different metabolic pathways under various environmental conditions (Staehelin, 2003). Based on our UV-B radiation treatments, there were different regulatory mechanisms in male and female *S. thunbergii*. Under low-intensity UV-B radiation, females depend on total amino acids. Under high-intensity UV-B radiation, males depend on soluble sugars.

### Differing molecular, enzymatic, and metabolic regulation response to UV-B radiation in dioecious *S. thunbergii*

**Figure 7:**
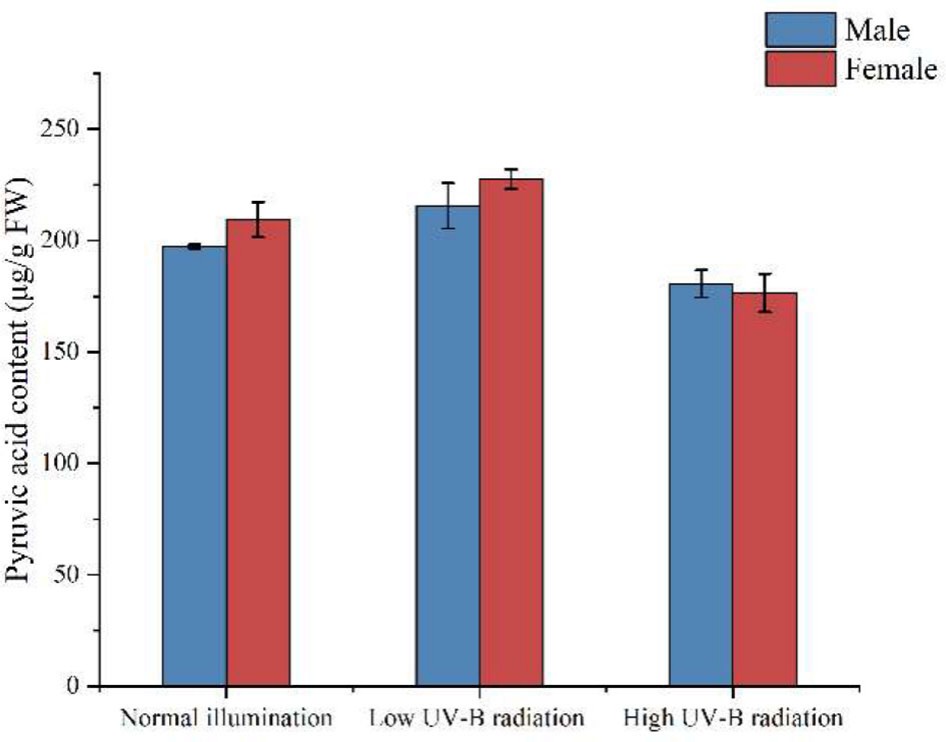
The amount of pyruvate in male and female *S. thunbergii* under different conditions.

Under UV-B radiation enhancement, differences between male and female *S. thunbergii* were observed at physiological levels as well as in gene expressions and enzymatic activity regulation. For example, in land plants, a series of amino acid metabolism related genes were upregulated in the male *Populus cathayana* but inhibited in the female (Zhang et al., 2014). This kind of regulation has also been observed in dioecious *S. thunbergii*. The RT-qPCR analysis shows that males have greater up regulation of genes than females. The carbon fixation genes *rubisco*, *ppdk*, and *fbp* were suppressed in the females under high-intensity UV-B radiation. As the key C_4_-related enzyme (Wang et al., 2013), PPDK activity and its transcriptional profile in brown macroalgae have the best correlation to environmental inorganic carbon concentrations and light intensities (Shao et al., 2019) and in carbon fixation processes, PPDK could catalyze the pyruvate forming phosphoenolpyruvate (Chi et al., 2014). In **Figure 7**, our results show no significant difference in pyruvate content between males and females under high-intensity UV-B radiation. The decrease in PPDK transcription affected the biosynthesis of carbohydrate and influenced the soluble sugar content in female macroalgae (**Table 2**). In contrast, the males maintained the same level of PPDK transcription and carbon fixation metabolite accumulation. PPDK can dissipate excess light energy. Decreasing PPDK activities in females might indicate that their redundant photorespiration process been enhanced and the Calvin cycle has been weakened. Compared to the males, the females showed more sensitivity to UV-B stress, which led to differences in the stress resistance of these dioecious macroalgae.

Pyruvate can also be catalyzed from G3P to generate acetyl-CoA, which connects the carbon fixation and TCA pathways. In these biochemical reactions, pyruvate can be catalyzed by PDH, forming acetyl-CoA, and by CS, forming citrate. The activity of PDH was slightly inhibited under low- and high-intensity UV-B radiation, but there was no significant difference between males and females in the three treatments. CS enzymes played an important role in the response of male microalgae to high-intensity UV-B radiation. Under high UV-B radiation, higher CS activities increased the citrate content in males (**Table 1**) and also provided more intermediate products to the next TCA pathways. Interestingly, our metabolomic analysis showed that in the control groups and low UV-B radiation treatment groups, the amount of citrate in males was significantly lower than in females. However, the males citrate content approached to females attatching with the citrate synthase activities more significantly up-regulated under high UV-B radiation, which indicated citrate and citrate synthase might be the key metabolite and enzyme participating in UV-B stressed regulation in males *S. thunbergii*. The gene expression profiles showed that *cs* had higher transcription levels in male algae than in females in the low and high-intensity UV-B radiation treatments, indicating that the transcription regulation of citrate synthase coding genes had been completed in males.

Glycolysis and pentose phosphate metabolism related genes were stimulated by UV-B radiation. Their expressed quantities increased, which could have resulted in post-transcriptional regulation. In male *S. thunbergii*, the expression of SODH transcription (*sodh*) remained slightly increased under low-intensity UV-B radiation, but under high-intensity UV-B radiation, it increased by 5.08 times compared to the control group. Under high-intensity UV-B radiation, D-sorbitol was 2.43 times higher in males than in females. This transcriptional regulation influenced the carbon-based metabolites, which also means that in male *S. thunbergii*, D-sorbitol biosynthesis served as an important process for adapting to UV-B stress. In addition, D-sorbitol reacts as the key metabolite in plant stress resistance (Tarczynski et al., 1993; Jennings et al., 1998) and research has identified sorbitol as a small molecular osmotic substance in land plants (Bellaloui et al., 1999), which could improve the ability of plants to resist cold, drought, salt, and toxic substance stresses. Our results found that D-sorbitol is a key metabolite used by male *S. thunbergii* to resist UV-B radiation.

The correlation analysis for photosynthetic ETR_max_, CCCM genes expression, enzyme activities, and key metabolites content could provide us with a new perspective (**Table S2**). The amount of soluble sugar and amino acids in males did not correlate well with photosynthetic physiological parameters, gene expression, or enzymatic activities. However, the amount of soluble sugar and amino acids in females showed a significant correlation with *Rubisco*, *sodh* expression and CS, PDH activities. This indicates that the amount of soluble sugar and amino acids in males is in a stable state. Even the stimulation of UV-B radiation did not change the expression of the CCCM genes and enzymatic activities, thus ensuring relatively stable soluble sugar and amino acids supply in males. The females were more susceptible to external stimuli, and initiate the transcription and enzymatic regulation of synthetic metabolites in response to UV-B stress. Correlation analysis could accurately reflect the relationships between physiological, molecular, and biochemical levels in male and female individuals. In terms of the amount of metabolites, when both genders are included only the expression of *acetyl-CoA* genes was closely related to the amount of soluble sugars, amino acids, and lipids (**Table S2C**). Dividing into different genders, the expression of *sodh* in males (**Table S2A**) and *Rubisco* in females (**Table S2B**) were shown to be significantly correlated with lipid content.

## Conclusion

Parameters involved in photophysiological ETR_max_ were measured to compare gender differences in *S. thunbergii* tolerance to UV-B. Males maintained their ETR_max_ values to a significantly greater extent than females under both low- and high-intensity UV-B radiation. Metabolomic results showed that the CCCM in *S. thunbergii* was significantly altered by UV-B radiation stress and that there were obvious gender differences. Under UV-B radiation stress, the expression of six targeted CCCM genes and the CS activity were significantly higher in males than in females. The amounts of typical carbon-based substances (soluble sugars, total amino acids, and lipids) showed that soluble sugars and amino acids served as the regulated metabolites in male *S. thunbergii* responding to UV-B radiation. The lipid accumulation was a key regulatory process in the response of females to UV-B radiation stress. Overall, these results provide deeper insights into the environmental stress tolerance mechanisms of male and female *S. thunbergii*, which is helpful for evaluating gender differences in the response of the brown macroalgae to UV-B radiation in the intertidal zone.

## Data availability statement

The original contributions presented in the study are included in the article/**Supplementary Material**. Further inquiries can be directed to the corresponding authors.

## Author contributions

YS conceived and designed the experiments, performed the experiments, analyzed the data, contributed reagents/materials/analysis tools, prepared figures and/or tables, authored or reviewed drafts of the paper, and approved the final draft. YZ and SS performed the experiments, analyzed the data, contributed reagents/materials/analysis tools, prepared figures and/or tables, authored or reviewed drafts of the paper, and approved the final draft. JC and JW analyzed the data, contributed reagents/materials/analysis tools, authored or reviewed drafts of the paper, and approved the final draft. QL and XT conceived and designed the experiments, analyzed the data, contributed reagents/materials/analysis tools, authored or reviewed drafts of the paper, and approved the final draft. All authors contributed to the article and approved the submitted version.

## Funding

This research was funded by the NSFC-Shandong Joint Fund (U1806213), National Natural Science Foundation of China (42176154) and the National Key R&D Program of China (2019YFD0901204).

## Acknowledgments

We thank LetPub (www.letpub.com) for its linguistic assistance during the preparation of this manuscript.

## Conflict of interest

The authors declare that the research was conducted in the absence of any commercial or financial relationships that could be construed as a potential conflict of interest.

## Publisher’s note

All claims expressed in this article are solely those of the authors and do not necessarily represent those of their affiliated organizations, or those of the publisher, the editors and the reviewers. Any product that may be evaluated in this article, or claim that may be made by its manufacturer, is not guaranteed or endorsed by the publisher.
