# Supplementary material for: The differing responses of central carbon cycle metabolism in male and female Sargassum thunbergii to ultraviolet-B radiation
Source: Front Plant Sci. 2022 Oct 3;13:904943. doi: 10.3389/fpls.2022.904943 (PMC9574197; doi:10.3389/fpls.2022.904943)
Supplement: Supplementary file 1 [file Table_1.docx]

Table S1: CCCM genes primers used in this study

| Genes | Code Number | F/R(5’-3’) | Sequence |
| --- | --- | --- | --- |
| *ribulose-l,5-bisphosphate carboxylase/oxygenase* (*Rubisco/Rbcl*) | Unigene0019872 | F | TGCGTATTGGGAGTTATGGG |
|  |  | R | GAAACCAGGCTCTTCGGCA |
| *fructose-1,6-bisphosphatase* (*fbp*) | Unigene0019476 | F | GCAGGAGGAGTGTATCGTGG |
|  |  | R | TCAGGAGCATTCGCTTCGTT |
| *pyruvate phosphodikinase* (*ppdk*) | Unigene0005129 | F | CCCGTATCTTCCTCAATCGCC |
|  |  | R | CGGCTTTGGTTCTCAACTTTCC |
| *citrate synthase*  (*cs*) | Unigene0047402 | F | ACCCGAAGGTGTCTGTGATG |
|  |  | R | CGTGTCTGGCTCTCTGGAAC |
| *succinic dehydrogenase*  (*sdh*) | Unigene0028161 | F | CTTGTCGTGAGGGCATCTGT |
|  |  | R | TAGTCGCTCCTGGGTGTAGT |
| *acetyl-CoA carboxylase*  (*ACC*) | Unigene0058167 | F | GTCATCCTCGTGTCCAGCAA |
|  |  | R | AGCAGTCGGGGGTCTTATCT |
| *sorbitol dehydrogenase sodh*  (*sodh*) | Unigene0062767 | F | ATCGGGGAGGTAGGAGGTTG |
|  |  | R | GGCTTTGTAGGGATGCGAGA |

The code number obtained from our previous transcriptomic database.
