# Supplementary material for: The differing responses of central carbon cycle metabolism in male and female Sargassum thunbergii to ultraviolet-B radiation
Source: Front Plant Sci. 2022 Oct 3;13:904943. doi: 10.3389/fpls.2022.904943 (PMC9574197; doi:10.3389/fpls.2022.904943)
Supplement: Supplementary file 2 [file Table_2.docx]

Table S2 Pearson’s correlation coefficients among different parameters in the male, female and dioecious-combined *S. thunbergii.* And the ** indicated significant correlation at the *p* < 0.01 level.

| **(A) Male** | | [**Photosynthsis**](javascript:;) | | | **Gene expression** | | | **Enzymic activity** | | **Metablites** | | | |
| --- | --- | --- | --- | --- | --- | --- | --- | --- | --- | --- | --- | --- | --- |
|  |  | **ETR_max_** | | | ***Rubisco*** | ***acetyl-CoA*** | ***sodh*** | **PDH** | **CS** | **soluble sugar** | **amino acids** | **lipid** | **pyruvate** |
| **ETR_max_** | | 1 | | | -0.572 | -0.988^**^ | -0.635 | 0.917^**^ | -0.865^**^ | 0.516 | -0.658 | 0.474 | 0.041 |
| ***Rubisco*** | |  | | | 1 | 0.555 | -0.200 | -0.294 | 0.773^*^ | -0.400 | -0.002 | 0.437 | 0.696^*^ |
| ***acetyl-CoA*** | |  | | |  | 1 | 0.658 | -0.931^**^ | 0.879^**^ | -0.422 | 0.606 | -0.489 | -0.102 |
| ***sodh*** | |  | | |  |  | 1 | -0.765^*^ | 0.377 | -0.085 | 0.619 | -0.939^**^ | -0.756^*^ |
| **PDH** | |  | | |  |  |  | 1 | -0.753^*^ | 0.298 | -0.642 | 0.687^*^ | 0.254 |
| **CS** | |  | | |  |  |  |  | 1 | -0.264 | 0.266 | -0.137 | 0.220 |
| **soluble sugar** | |  | | |  |  |  |  |  | 1 | -0.487 | 0.058 | -0.243 |
| **amino acids** | |  | | |  |  |  |  |  |  | 1 | -0.671^*^ | -0.335 |
| **lipid** | |  | | |  |  |  |  |  |  |  | 1 | 0.818^**^ |
| **pyruvate** |  | | |  | |  |  |  |  |  |  |  | 1 |
| **(B) Female** | | | **ETR_max_** | ***Rubisco*** | | ***acetyl-CoA*** | ***sodh*** | **PDH** | **CS** | **soluble sugar** | **amino acids** | **lipid** | **pyruvate** |
| **ETR_max_** | | | 1 | 0.679^*^ | | -0.980^**^ | -0.954^**^ | 0.961^**^ | -0.033 | 0.331 | -0.690^*^ | 0.518 | 0.675^*^ |
| ***Rubisco*** | | |  | 1 | | -0.794^*^ | -0.453 | 0.613 | 0.694^*^ | 0.901^**^ | 0.048 | 0.957^**^ | 0.954^**^ |
| ***acetyl-CoA*** | | |  |  | | 1 | 0.891^**^ | -0.950^**^ | -0.146 | -0.479 | 0.565 | -0.648 | -0.799^**^ |
| ***sodh*** | | |  |  | |  | 1 | -0.951^**^ | 0.299 | -0.070 | 0.847^**^ | -0.262 | -0.465 |
| **PDH** | | |  |  | |  |  | 1 | -0.062 | 0.274 | -0.715^*^ | 0.424 | 0.679^*^ |
| **CS** | | |  |  | |  |  |  | 1 | 0.893^**^ | 0.720^*^ | 0.753^*^ | 0.675^*^ |
| **soluble sugar** | | |  |  | |  |  |  |  | 1 | 0.427 | 0.927^**^ | 0.851^**^ |
| **amino acids** | | |  |  | |  |  |  |  |  | 1 | 0.204 | 0.000 |
| **lipid** | | |  |  | |  |  |  |  |  |  | 1 | 0.886^**^ |
| **pyruvate** | | |  |  | |  |  |  |  |  |  |  | 1 |
| **(C) Male and female** | | **ETR_max_** | | ***Rubisco*** | | ***acetyl-CoA*** | ***sodh*** | **PDH** | **CS** | **soluble sugar** | **amino acids** | **lipid** | **pyruvate** |
| **ETR_max_** | | 1 | | 0.196 | | -0.863^**^ | -0.820^**^ | 0.394 | 0.393 | 0.423 | -0.230 | 0.148 | 0.395 |
| ***Rubisco*** | |  | | 1 | | 0.130 | -0.535^*^ | 0.586^*^ | -0.100 | 0.441 | 0.437 | -0.048 | 0.452 |
| ***acetyl-CoA*** | |  | |  | | 1 | 0.445 | -0.125 | -0.381 | -0.340 | 0.487^*^ | -0.505^*^ | -0.522^*^ |
| ***sodh*** | |  | |  | |  | 1 | -0.681^**^ | -0.226 | -0.279 | -0.086 | 0.275 | -0.199 |
| **PDH** | |  | |  | |  |  | 1 | -0.315 | -0.155 | -0.031 | -0.209 | 0.151 |
| **CS** | |  | |  | |  |  |  | 1 | 0.171 | -0.378 | 0.291 | 0.284 |
| **soluble sugar** | |  | |  | |  |  |  |  | 1 | 0.407 | 0.254 | .482^*^ |
| **amino acids** | |  | |  | |  |  |  |  |  | 1 | -0.545^*^ | -0.235 |
| **lipid** | |  | |  | |  |  |  |  |  |  | 1 | 0.761^**^ |
| **pyruvate** | |  | |  | |  |  |  |  |  |  |  | 1 |
